# Acute Cerebral Artery Occlusion Following Levantine Viper (*Macrovipera lebetina*) Bite: Gács Sign in Snakebite

**DOI:** 10.34172/aim.31131

**Published:** 2025-04-01

**Authors:** Seyed Mostafa Mirakbari, Reza Gorji

**Affiliations:** ^1^Clinical Research Development Unit, Department of Clinical Toxicology, Bu Ali Hospital, Qazvin University of Medical Sciences, Qazvin, Iran; ^2^Clinical Research Development Unit, Department of Neurology, Bu Ali Hospital, Qazvin University of Medical Sciences, Qazvin, Iran

**Keywords:** Anti-snake venom, Cerebral stroke, Poisonous snake, Snakebite, Snake envenoming

## Abstract

Acute cerebral thrombotic stroke following a viper snakebite is a rare occurrence. There have been a few documented cases of cerebral infarctions resulting from envenomation by various viper species. However, none of these reports have specifically detailed instances of stroke induced by *Macrovipera lebetina* envenomation or vessel occlusion without concurrent cerebral infarction. In this study, we present a case of middle cerebral artery (MCA) occlusion in a 56-year-old man who was bitten by a *Macrovipera* or *Vipera lebetina* snake. The individual experienced a snakebite on his right foot, leading to subsequent seizure activity and loss of consciousness. Non-contrast computed tomography scan of the brain revealed hyperattenuation of the right MCA, indicating clot occlusion within the artery, commonly referred to as hyperdense MCA sign or Gács sign.

## Introduction

 Snakebites represent a significant health hazard in tropical and subtropical regions. In Iran, snakebites occur at an estimated rate of 7.42 per 100 000 individuals annually, with viper species being the primary culprits.^1^ Viper bites typically present with local cellulitis, renal failure, and coagulopathies. However, thrombotic complications following snakebites are infrequently documented, and our knowledge of the neurological consequences of viper envenomation remains limited.^2,3^ In this report, we detail the case of an individual bitten by a viper snake (*Macrovipera lebetina* or *Vipera lebetina*), who exhibited symptoms of seizure, loss of consciousness, and local manifestations at the bite site upon presentation to the emergency department. Non-contrast computed tomography of the brain revealed the presence of the dense middle cerebral artery (MCA) sign (also known as Gács sign) without any discernible cerebral infarction. The importance of addressing this snakebite case is twofold: firstly, it is critical due to the thrombotic cerebral consequence; secondly, due to the occurrence of a seizure event, both of which are quite rare in viper snakebites.

## Case Report

**Figure 1:**
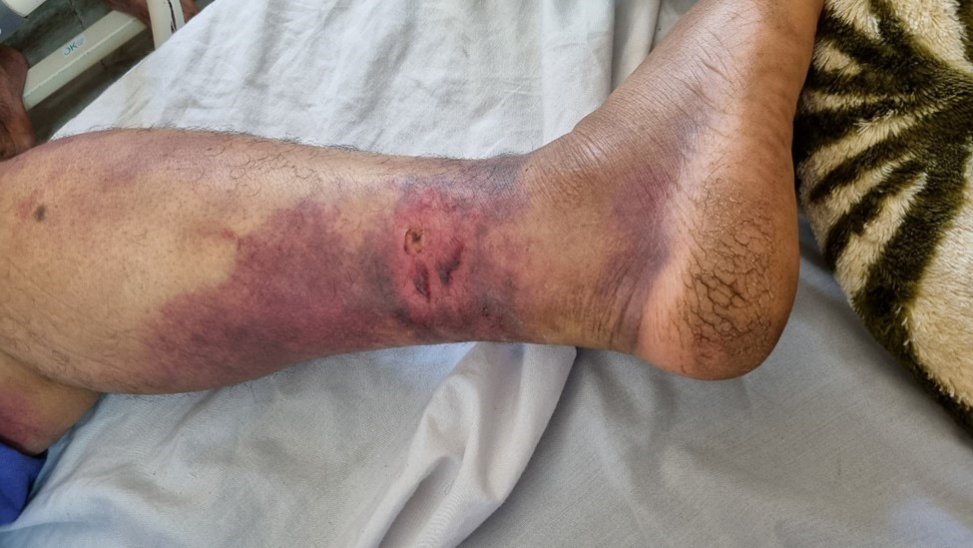
Fang Punctures, Two Blisters and Bruise Following Viper Snake Bite (*Vipera lebetina*)

**Figure 2:**
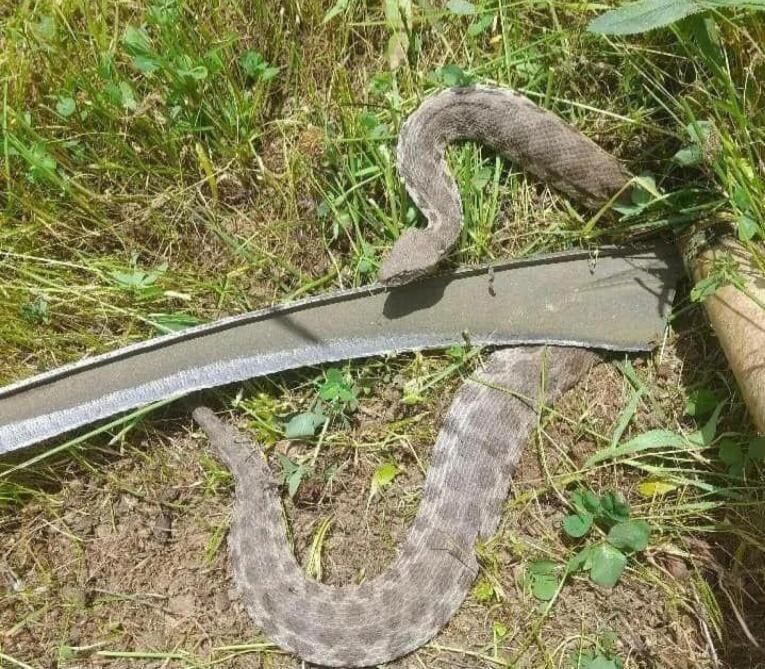
*Vipera lebetina* in Iran (10 July 2023), by @gilan_eshkevar, available at https://www.instagram.com/p/Cug1DGVNpUR/?igsh=MTl0eGk2dXh0ZmtxZg==, unchanged from original image.

 A 56-year-old previously healthy man was bitten by a snake on the lateral aspect of his right foot while working on the farm (Figure 1). The snake, which was killed and brought to the emergency department by locals, was identified by a clinical toxicologist as the ‘Gorzeh Mar’ in the Persian glossary, which is equivalent *to M. lebetina* or *V. lebetina* (Figure 2). The patient’s companions noticed that after the bite, he experienced severe pain accompanied by general body numbness, leading to a feeling of faintness. They immediately called emergency medical services. During the transfer and one hour after the bite, he had a generalized seizure, characterized by clenching his jaw tightly, frothing, and rigidity of limbs, which was controlled by administering 3 mg of midazolam. The choice of midazolam for managing seizure event was preferred due to its short duration of sedative action, which minimizes interference with subsequent neurological examinations that are essential for determining the appropriate antivenom therapy.

 Upon admission, he was unconscious and unresponsive to verbal commands. He reacted to central painful stimuli by moaning and posturing. His vital signs were recorded as follows: blood pressure 100/85 mm Hg, pulse rate 110 beats·min^-1^, temperature 36.9 °C, and respiratory rate 10 breaths·min^-1^. Initial emergency investigations found sinus tachycardia on the electrocardiogram, an oxygen saturation of 94% with mask oxygen on pulse oximetry, and metabolic acidosis with a pH of 7.19 on arterial blood gas analysis. The blood test results showed leukocytosis, thrombocytopenia, and prolonged prothrombin and partial thromboplastin time.The laboratory results are detailed in Table 1. A history of snakebite reported by relatives, along with two punctured fang marks on the foot associated with bleeding, indicated a diagnosis of snakebite (Figure 1). Based on the clinical manifestations and laboratory findings outlined in the viper snakebite envenomation severity scale (SESS), a classification of very severe toxicity grade was established.^4^ SESS was developed and enhanced by efforts of Monzavi et al to grade envenomation severity based on clinical manifestations and lab findings.^4^ According to the algorithm for management of snakebite envenomation in Iran, the patient promptly received 10 vials of Razi^TM^ Polyvalent Antivenin within one hour of admission.^4^ The patient was immediately intubated and transferred to the intensive care unit.

**Table 1:** Blood Biochemical Tests of the Patient

**Test**	**Result**	**Unit**	**Reference Values**
White blood cell	8	10^3^/µL	4-10
Hemoglobin	13	g/dl	14-17.5
Platelet	136	10^3^/µL	172-450
Aspartate transferase	32	mg/dL	Up to 37
Alanine transferase	20	mg/dL	Up to 41
Alkaline phosphatase	86	mg/dL	64-306
Creatinine phosphokinase	163	mg/dL	24-195
Lactate dehydrogenase	376	mg/dL	480˂
Bilirubin total	0.7	mg/dL	0.1-1.2
Bilirubin direct	0.2	mg/dL	0.25 ≥
Blood urea nitrogen	19	mg/dL	7-21
Creatinine	1.2	mg/dL	0.7-1.4
Sodium	139	mEq/L	136-146
Calcium	8.9	mEq/L	8.5-10.5
Prothrombin time	43.3	seconds	13-15
International normalized ratio	3.33	-	1
Partial thromboplastin time	120	seconds	25-40

**Figure 3:**
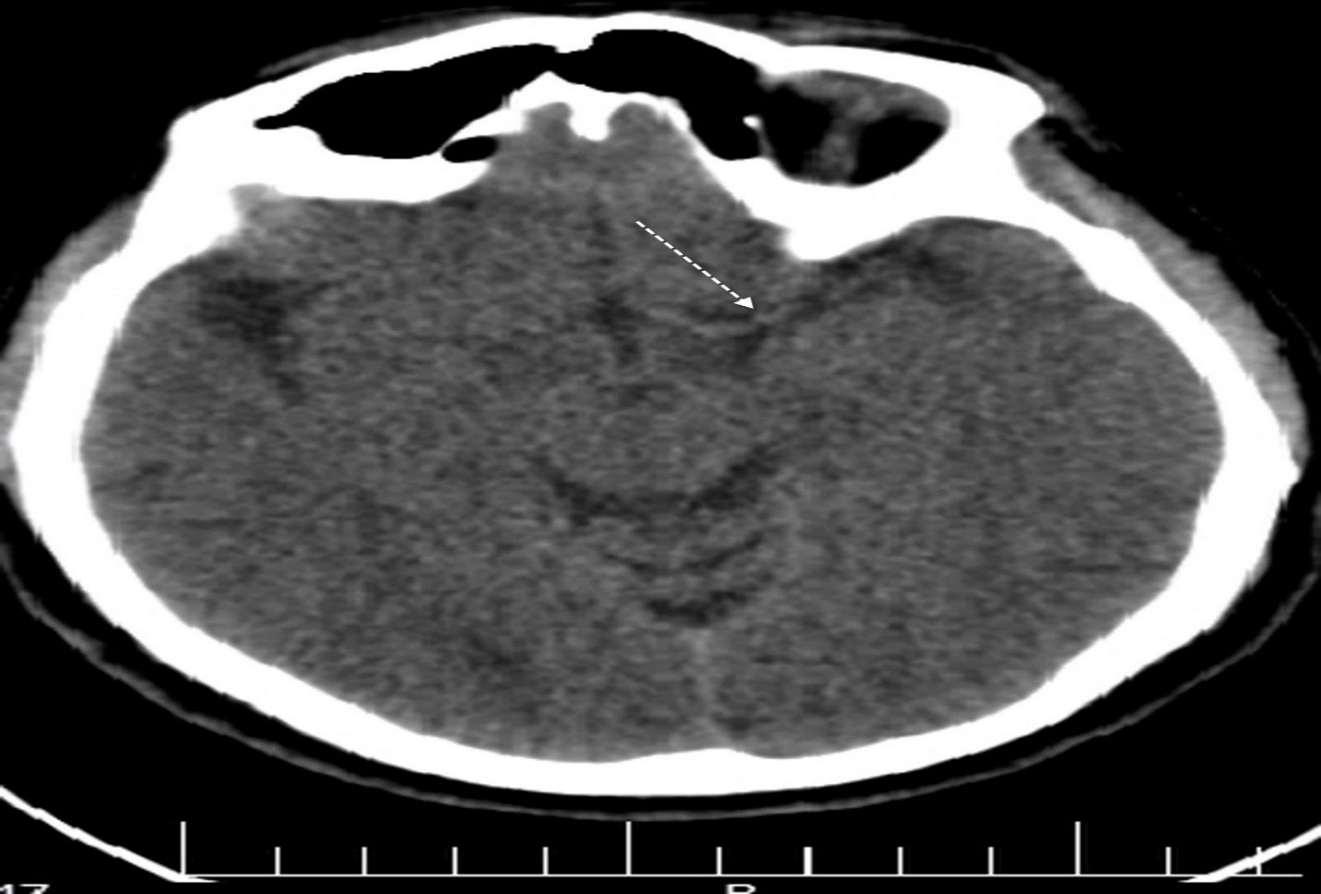
Non-contrast Computed Tomography Scan of the Brain Showing Left Dense Middle Cerebral Artery Sign (Also Known as Gács sign) in Patient with *Vipera lebetina* Envenoming

 The patient was rehydrated with infusion of 1000 mL 0.9% sodium chloride (normal saline) within one hour, followed by maintenance infusion of 2500 mL over the next 24 hours. A single vial of tetanus toxoid was administered, and combination antibiotic therapy with ciprofloxacin and clindamycin was initiated. A non-contrast brain tomography scan performed 6 hours after admission revealed hyperattenuation of the MCA, suggestive of an occluding clot in the artery (Figure 3). Five vials of polyvalent antivenom were re-administered 12 hours later in accordance with guideline.^4^ Twenty-four hours following the snakebite (day 2), the patient regained consciousness and was extubated. Care was continued for the subsequent 24 hours. By day 3, neurological examination yielded unremarkable results. Muscle strength in the upper and lower extremities measured at 5/5. Repeat computed tomography was not performed as the patient’s recovery was progressing rapidly. On day 4, the patient was ambulating without assistance. No signs of clumsiness or loss of balance were observed. He was discharged home on day 6 with normal laboratory results and in good general condition.

## Discussion

 Ischemic stroke occurrences following snakebites are scarcely documented in the existing literature. While arterial thrombotic complications have been noted in cases of *Viperidae* family envenomation, such occurrences have not been attributed to bites from the Levant blunt-nosed viper*, M. lebetina*.^2,3,5-12^ The processes that lead to cerebral infarction during viper venom exposure can involve multiple factors. The viper venom exhibits both anticoagulant and procoagulant properties; the procoagulant and platelet-aggregating effects are attributed to compounds such as cerastobin, factor IVa, cerastocytin, cerastotin, and afaacytin.^13-15^ These various protein components exhibit thrombin-like enzymatic activity; different toxins target various aspects of the coagulation cascade, leading to fibrin formation in the bloodstream, which can cause small or large vessel blockages due to micro-thrombi, potentially resulting in cerebral infarction, or the toxin may induce severe vasospasm. Additionally, haemorrhagins are toxic components found in snake venom that are activated by the complement system. These elements can induce severe vascular spasms, damage the endothelial lining of blood vessels, and increase vascular permeability. Collectively, these effects may lead to toxic vasculitis, which can ultimately result in thrombosis.^16,17^ Also, hypotension may result from hypovolemia caused by sweating, vomiting, reduced fluid intake, and bleeding, which can lead to low flow states and watershed infarcts. Additionally, hyperviscosity from hypovolemia and hypoperfusion due to hypotension and hypercoagulation can contribute to vessel occlusion.^3^ Although, the mechanisms underlying blood clot formation and vascular occlusion associated with viper bites are well-documented, there is a paucity of information regarding the pathophysiology of convulsions in vipers, particularly in *M. lebetina*. Typically, elapid venoms contain toxins that influence the nervous system and are classified as neurotoxic, whereas viperid venoms primarily target blood coagulation and are considered hemotoxic. Most viperine snakebites are hemotoxic. Although rare, dual neurotoxic symptoms can occur following a viperine bite, mostly in the case of Russell’s viper. In a study conducted by Anjana Silva *et al.* involving 245 confirmed cases of Russell’s viper bites, 68% exhibited coagulopathy and 53% showed signs of neurotoxicity, characterized by ptosis (100%), blurred vision (93%), and ophthalmoplegia (90%).^18^ Lahiri et al documented a case of status epilepticus following a viper bite, emphasizing that such a presentation is exceedingly rare.^19^ The exact cause of neurotoxicity in Russell’s viper is not fully understood, but phospholipase A2 (PLA2) is believed to play a major role. This toxin alters the lipid bilayer of nerve cell membranes, destabilizing them and hindering the fusion of synaptic vesicles and the effective release of neurotransmitters. Helicopsin, found in viper *Bothrops alternatus*venom is categorized as a neurotoxin based on neurological signs characteristic of depolarizing neuromuscular blocking neurotoxins. These signs include edginess, ataxia, convulsions, flaccid paralysis of respiratory muscles, and death.^20^

 Nevertheless, the occurrence of seizure events following bites from *M. lebetina* has not been previously reported. Kazemi et al documented five cases of individuals bitten by *M. lebetina* in Iran, showcasing a range of manifestations that resulted in musculoskeletal disabilities.^21^ However, none of these cases exhibited involvement of the cerebral arteries or experienced seizures. Sharma et al have recently shed light on the Levantine viper (*M. lebetina*), a species with limited documentation in India.^22^ They detailed a case of a 33-year-old male bitten by *M. lebetina*, who subsequently displayed local edema, bruising, and necrosis around the bite site, with no indications of neurological impairment.

 We searched the literature to elucidate the underlying reasons for the seizure observed after *M. lebetina* bites, a phenomenon not previously documented. We hypothesize that this seizure event could be linked to the presence of Phospholipases type A2 in *M. lebetina* venoms.^23^ These enzymes are among the most prevalent proteins in the venom and are known to possess neurotoxic properties.^24^ The association between specific toxic secreted phospholipases A2 and their potential to trigger epileptic seizures has sparked continued interest in exploring their mechanisms of action and their impact on neurological functions. In our case, two potential mechanisms for the onset of seizures may be proposed. The first involves swift emergence of cortical hypoxia due to MCA clot obstruction. The second mechanism pertains to the inherent seizure-inducing properties of the venom itself.

 Our patient experienced an atypical recovery from a thrombotic event. The reversal of an acute ischemic stroke in the MCA region, caused by a viper snakebite, is a rare occurrence. Sahoo and Sriramka reported this case but were unable to identify the specific snake subtype.^7^ The patient demonstrated gradual improvement following early and continuous administration of anti-snake venom (ASV), which was administered within the first 6 hours post-bite. Timely ASV administration is crucial to prevent the venom from becoming fixed in tissues, as suggested by previous research.^4,6^

 The first report of a hyperdense cerebral artery in the context of acute ischemic stroke was documented by Gács et al in 1983.^25^ The visibility of a hyperdense cerebral artery serves as an early indicator, appearing well before the occurrence of parenchymal changes commonly referred to as early ischemic signs.^26^ This visual manifestation becomes apparent when a cerebral blood vessel experiences occlusion. Recognizing this early sign is crucial in the diagnosis and management of ischemic strokes, as it allows for prompt intervention and treatment. By identifying the hyperdense cerebral artery, healthcare professionals can initiate appropriate measures to mitigate the impact of the occlusion and potentially prevent further damage.

## Conclusion

 Our case presents an uncommon clinical manifestation of *M. lebetina* snakebite, as there has been no prior report of vascular clot occlusion and seizure subsequent to this type of envenomation. Early administration of snake antivenom, in conjunction with maintaining hemodynamic stability, played a significant role in preventing further cerebral ischemic infarction. Future studies should focus on elucidating the pathophysiological mechanisms underlying seizure events and cerebral occlusion following *M. lebetina* bites.
